# The effect of coffee on contralateral suppression of transient evoked otoacoustic emissions

**DOI:** 10.12688/f1000research.122851.1

**Published:** 2022-08-02

**Authors:** Ishaan Srivastava, Mohan Kumar Kalaiah, Ritik Roushan, Usha Shastri, Kaushlendra Kumar

**Affiliations:** 1 Department of Audiology and Speech Language Pathology, Kasturba Medical College, Mangalore, Manipal Academy of Higher Education, Manipal, India

**Keywords:** Otoacoustic emissions, TEOAE, Contralateral suppression, Coffee, Caffeine

## Abstract

**Background**: Coffee is a popular non-alcoholic beverage consumed by humans across the world. It contains caffeine, which is a type of stimulant of the central nervous system. In the auditory system, it has a positive effect on auditory brainstem response and perception of speech in noise. Further, caffeine has an inhibitory effect in the cochlea, but studies have rarely investigated its effect on otoacoustic emissions (OAEs) in humans. OAEs are low-intensity sounds produced by the cochlea, which could be recorded in the ear canal. The present study was carried out to investigate the effect of coffee on transient evoked otoacoustic emission (TEOAE) and contralateral suppression of TEOAE.

**Method**: A total of 52 young adults participated in the study. A cross-over study design was used for the present investigation. The TEOAE and contralateral suppression of TEOAE were recorded before and after consumption of coffee and milk. The contralateral suppression of TEOAE was measured by presenting white noise to the contralateral ear at 40, 50, and 60 dB sound pressure level (SPL).

**Results**: The mean amplitude of TEOAE before and after consumption of coffee was similar in both ears. Further, the mean contralateral suppression of TEOAE was slightly larger after consumption of coffee in both ears. However, the mean difference was not significant in both the ears.

**Conclusions**: Based on the findings of present study, coffee has no significant effect on the amplitude of TEOAE and contralateral suppression of TEOAE.

## Introduction

Coffee is a non-alcoholic beverage which is widely consumed by humans across the world. ^1^^–^^3^ It contains a variety of bioactive chemicals that have anti-oxidant, anti-inflammatory, and anti-cancer properties. ^1^ It also contains caffeine which is a stimulating agent. Caffeine is also found in various other food items such as tea, cocoa beans, chocolate, energy drinks, among others. ^1^^,^^2^ Further, the amount of caffeine in any food product is determined by the serving size, product type, and preparation method. ^4^ Caffeine improves perception, increases the abilty to remain awake for longer periods, and reduces fatigue. ^5^ The stimulatory effect of caffeine is due to the blocking of adenosine receptors, consequently regulating the neurotransmitter levels and activities in the central nervous system. ^3^

Otoacoustic emissions (OAEs) are very small amplitude sounds produced by the cochlea as a by-product of motile function of the outer hair cells (OHCs) (i.e. amplifier function of OHCs). ^6, 7^ The OAEs generated in the cochlea travel backwards through the middle ear to the external ear canal, and it can be recorded using a sensitive microphone from the external ear canal. ^6– 8^ In the cochlea, the OAEs are produced spontaneously and also in response to an external acoustic stimuli, referred as spontaneous OAEs and evoked OAEs respectively. Further, the OAEs elicited in response to short-duration stimuli such as clicks and tone-bursts are known as transient evoked OAEs (TEOAEs). ^8^ The OAEs elicited in response to pure-tones are known as distortion product OAEs (DPOAEs) and stimulus frequency OAEs (SFOAEs).

The human auditory system comprises afferent and efferent auditory pathways. The efferent pathways have an inhibitory function in the auditory system. In the cochlea, the efferent fibres cause hyperpolarization of OHCs, subsequently reducing their motile function. ^9^ The reduced motility of OHCs in the presence of efferent activity result in a reduction of the amplitude of OAEs. ^10^ This reduction in the amplitude of OAE due to efferent activity is called suppression of OAE. In humans, the suppression of OAE can be measured by presenting noise to the test ear or non-test ear during the recording of OAEs. The suppression of OAE obtained by presenting noise to the non-test ear is known as the contralateral suppression of OAE.

Several studies have investigated the effect of caffeine on the auditory system. Studies have been carried out to understand the effect of caffeine on auditory evoked potentials, ^11^^–^^20^ speech perception, ^21^^,^^22^ and otoacoustic emissions. ^23^ Few studies have investigated the effect of caffeine on the auditory brainstem response (ABR). Findings from these investigations have showed significantly shorter latency and larger amplitude for ABR peaks following caffeine ingestion. ^11^^,^^12^^,^^16^ Similarly, caffeine has been found to have an effect on the middle latency response and late latency response. ^12^ In general, findings of the above studies suggest a positive effect of caffeine on the central auditory pathway. Very few studies have investigated the effect of caffeine on speech perception ability. ^21^^,^^22^ Altin *et al.* ^21^ investigated the effect of caffeine on speech identification score in noise. Results showed a significant improvement for speech identification score in noise after caffeine ingestion. Taghavi *et al.* ^22^ investigated the short-term effect of caffeine on the acceptable noise level (ANL) in individuals with normal hearing. The results showed a significant reduction in the ANL after caffeine intake, suggesting that caffeine increases tolerance to noise, improving speech perception in noise. Based on findings from the above investigations, caffeine could be assumed to have a positive effect on the perception of speech in noise.

Studies investigating the effect of caffeine on the OAEs in humans are scarce. Various studies investigating the effect of caffeine on the cochlea have reported, the caffeine causes hyperpolarization of OHCs in the cochlea which suppress the amplifier function of OHCs. ^24^^–^^26^ Therefore, caffeine could be assumed to have a negative effect on the amplitude of OAEs. Recently, Drepath *et al.* ^23^ reported no significant effect of coffee on the amplitude of DPOAE. In contrast, Bobbin ^27^ reported an effect of caffeine on the amplitude of DPOAE in animal study. Therefore, although Drepath *et al.* ^23^ reported no effect of coffee on the amplitude of OAE in humans; similar investigations should be conducted before generalizing the results. Thus, the first objective of the present study was to investigate the effect of coffee on the amplitude of TEOAE. The second objective of the present study was to investigate the effect of coffee on the contralateral suppression of TEOAE, to understand the effect of caffeine on efferent activity in the auditory system. Studies investigating the effect of caffeine on the ABR have reported an improved transmission of neural activity in the auditory pathway. This improved transmission in the afferent pathways could also elicit stronger activity in the efferent pathways. However, none of the studies have investigated the effect of caffeine of the efferent activity. Further, studies investigating the effect of caffeine on the speech perception have reported a positive effect of caffeine on perception of speech in noise. The improvement in speech perception after caffeine ingestion could be a consequence of increased efferent activity in the auditory system. Studies investigating the role of efferent activity on perception of speech in noise have reported a significant relationship between the magnitude of efferent activity and speech perception in noise. ^28^ Therefore, there is a need to understand the effect of caffeine on the efferent activity.

## Method

### Participants

A total of 52 adults (nine males, 43 females) aged between 19 and 24 years (mean=21.65, standard deviation (SD)=1.36) participated in the study. All participants had hearing sensitivity within normal limits in both ears. The pure-tone threshold was less than 15 dB HL at octave frequencies from 250 Hz to 8000 Hz. Immittance evaluation showed ‘A’ type tympanogram with acoustic reflex present at normal levels in both ears. None of the participants had a history of otological problems, such as ear pain, ear discharge etc. None of the participants reported exposure to loud sounds or ototoxic medication. Individuals who agreed to participate in the study were randomly assigned into two groups (coffee-first group and milk-first group) using drawing lots method. The acoustic reflex threshold for white noise was greater than 70 dB SPL for all the participants. The study was approved by the institutional ethics committee of Kasturba Medical College, Mangalore (Protocol number: IEC KMC MLR 03-2021/89) and informed consent was obtained from all the participants.

### Procedure

**Figure 1.**
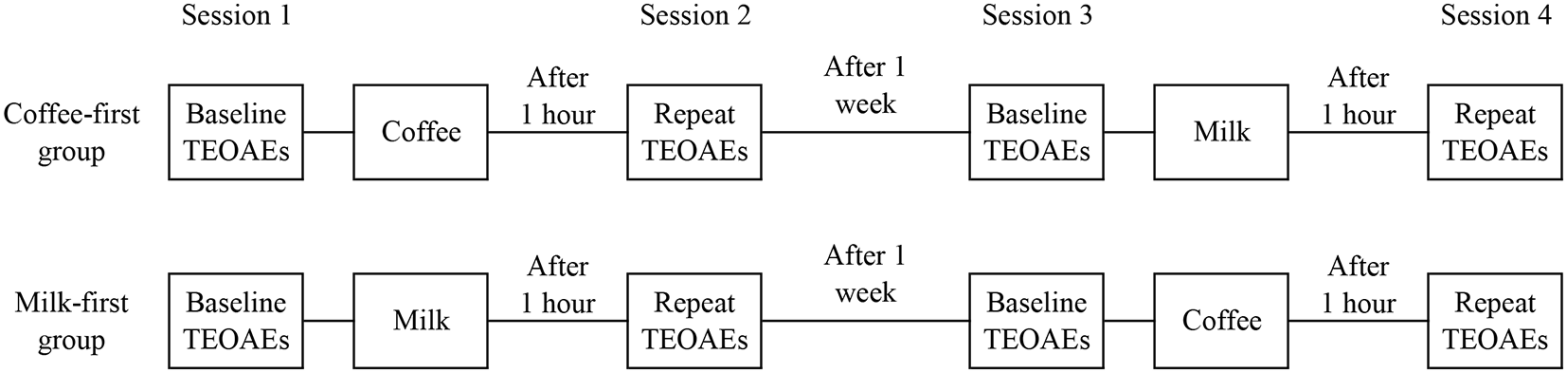
Schematic diagram showing the procedure followed for data collection.

Individuals who agreed to participate in the study were randomly assigned into two groups, namely the ‘coffee-first’ and ‘milk-first’ groups. The drawing lots method was used to assign the participants into two groups. The data collection was carried out in two phases. In phase I, the TEOAE and contralateral suppression of TEOAE were recorded before and after consumption of coffee (coffee-first group) or milk (milk-first group). In phase II, the TEOAE and contralateral suppression of TEOAE were recorded before and after consumption of milk (coffee-first group) or coffee (milk-first group). Phase II of the study was carried out after a gap of one week. Further, participants were informed to restrain from consuming caffeinated substances such as coffee, tea, energy drinks, or chocolate. for at least 12 hours prior to data collection. The procedure followed for data collection is shown in Figure 1.

*Recording of TEOAEs*

The TEOAEs were recorded using the Otodynamics Echoport 292II otoacoustic emission analyzer. During the recording of TEOAEs, participants were made to sit comfortably on the reclining chair. They were instructed not to move throughout the duration of recording of TEOAEs. The OAE probe was fitted to the test ear and E-A-RTone 5A insert phone was fitted to the contralateral ear of participants. Initially, the TEOAE was recorded in non-linear mode. A total of 260 click-trains (1040 clicks) were presented at 80 dB SPL, and the responses were averaged. Following this, the TEOAEs were recorded in linear-mode. A total of four recordings were obtained with and without presenting noise to the contralateral ear of participants. In each recording, a total of 400 click-trains (1600 clicks) were presented at 60 dB SPL and the responses were averaged. The first recording of TEOAE was always obtained without presenting noise to the contralateral ear, and referred to as baseline TEOAE. The remaining three recordings were obtained by presenting white noise to the contralateral ear of participants at 60 dB SPL, 50 dB SPL, and 40 dB SPL. The order of noise level presented to the contralateral ear was randomized. All the recordings of TEOAE were obtained without disturbing the placement of OAE probe ( *i.e.*, single-fit condition). Further, the TEOAEs were recorded from both ears of the participants.

The TEOAEs were recorded in four sessions. The first two sessions were scheduled on day 1 and the remaining two sessions were scheduled after one week. In the first session, the TEOAEs were recorded in non-linear and linear modes and these recordings were referred to as ‘baseline measurements’. After completing the baseline measurements, coffee was given to participants in the ‘coffee-first’ group and milk was given to participants in the ‘milk-first’ group. After one hour, the second session of TEOAE recording was initiated. The TEOAEs obtained in the second session were similar to the first session and were referred as ‘follow-up measurements’. After one week, the baseline (session 3) and follow-up (session 4) measurements were repeated. After the third session milk was given to participants in the ‘coffee-first’ group and coffee was given to participants in the ‘milk-first’ group.

*Coffee preparation*

One sachet of instant coffee powder (1.3 g – 70% coffee and 30% chicory) and two tablespoons of powdered milk were mixed in 150 mL of warm water and sugar was added to improve the flavour for each serving. Milk was prepared similarly without adding the coffee powder.

### Data analysis

The global amplitude of TEOAE and noise-floor level was computed using EchoMaster software. ^29^ The TEOAEs recorded in non-linear mode were considered to be present if the global signal-to-noise ratio (SNR) was at least 6 dB SNR. Further, the TEOAEs recorded in linear mode were considered to be present if the global SNR was at least 3 dB SNR. The magnitude of contralateral suppression of TEOAE was calculated by subtracting the global amplitude of TEOAE in various contralateral noise conditions ( *i.e.*, 60, 50 and 40 dB SPL) from the baseline condition.

## Results

### Transient evoked otoacoustic emissions

**Figure 2.**
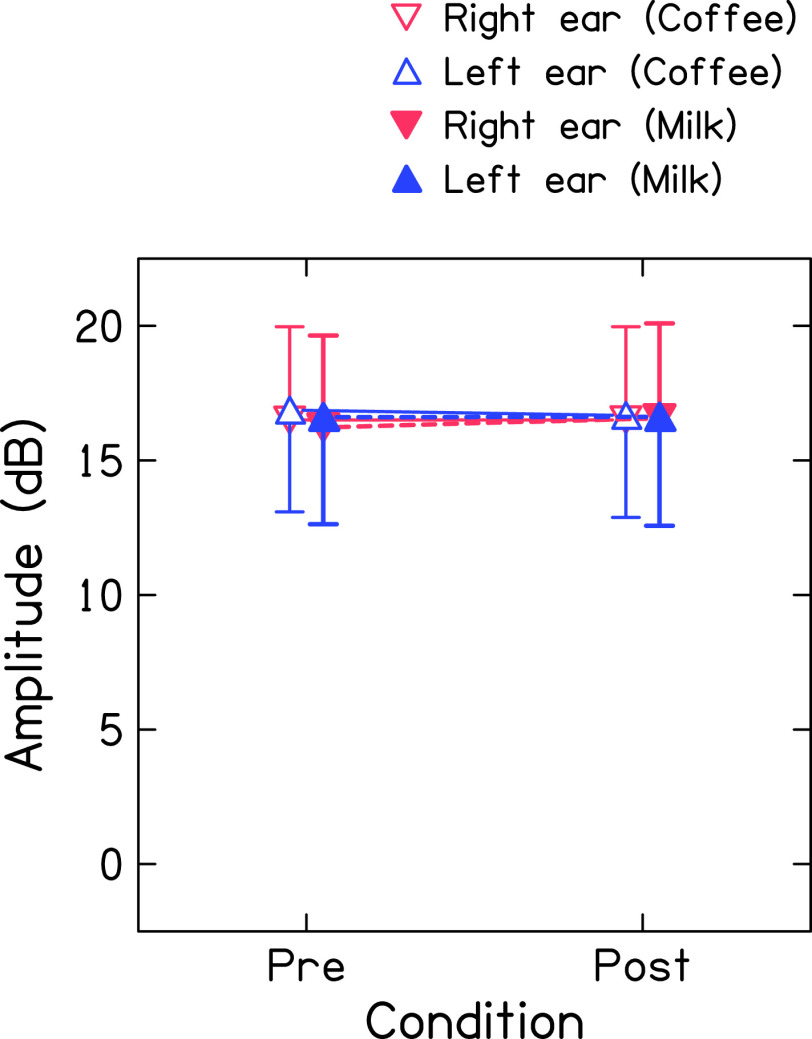
Mean global amplitude of TEOAE (recorded in non-linear mode at 80 dB SPL) before and after consumption of coffee (unfilled triangles connected with solid line) and milk (filled triangles with dashed line) for right ear (red colour) and left ear (blue colour).

Figure 2 shows the mean global amplitude of TEOAE (recorded in non-linear mode at 80 dB SPL) for both ears before and after consumption of coffee and milk. The mean amplitudes were similar for both ears across the conditions ( *i.e.*, before and after consumption of coffee and milk). The Shapiro-Wilk test revealed that the amplitude of TEOAE of both ears across conditions was normally distributed. Thus, a repeated-measures ANOVA was carried out with ears (right and left), conditions (before and after consumption), and drink (coffee and milk) as repeated measures. Results showed no significant effect of ear [F(1,42)=0.505, p=0.481], condition [F(1,42)=0.162, p=0.689], and drink [F(1,42)=0.644, p=0.427] on the amplitude of TEOAE. Further, no significant interaction was found between ears and conditions [F(1,42)=2.016, p=0.163], drink and conditions [F(1,42)=0.644, p=0.427], ears and drink [F(1,42)=0.09, p=0.765], and ears, drink, and conditions [F(1,42)=0.135, p=0.751]. Bayesian repeated measures-ANOVA showed moderate evidence in favour of the null hypothesis for the effect of conditions [BF _10_=0.127] and drink [BF _10_= 0.156] on the amplitude of TEOAEs. Further, it showed anecdotal evidence in favour of the null hypothesis for the effect of ears [BF _10_=0.366] on the amplitude of TEOAE, which suggests more data need to be collected to draw a firm conclusion (Dienes, 2014).

**Figure 3.**
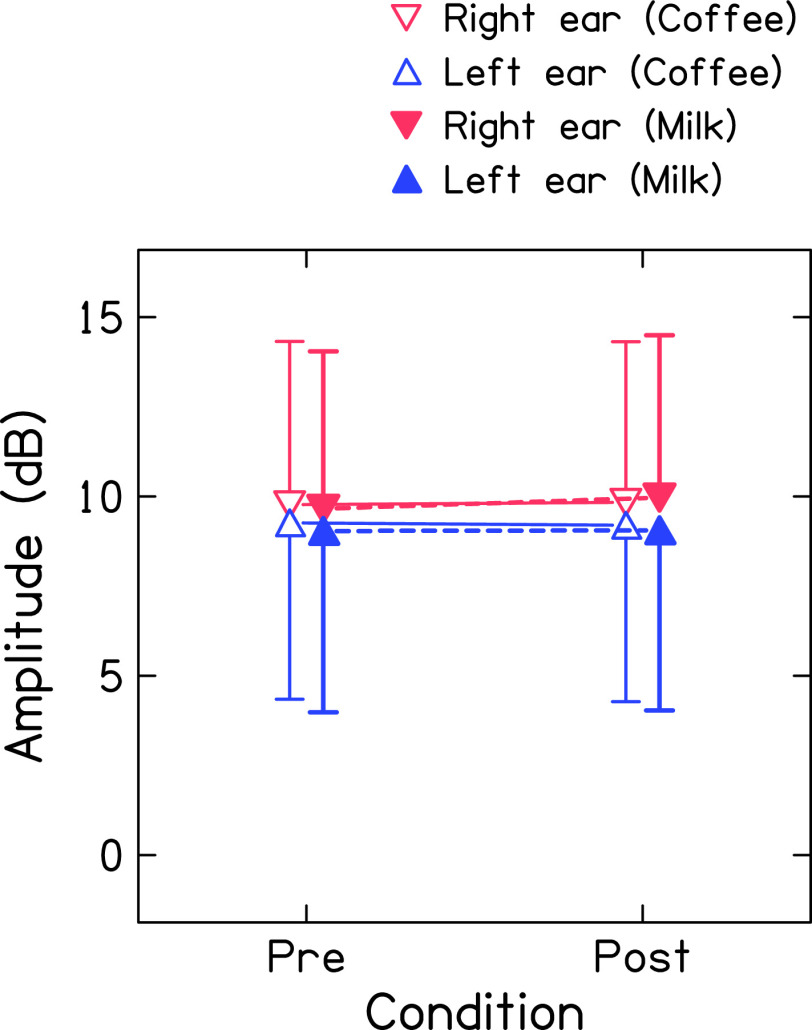
Mean global amplitude of TEOAE (recorded in linear mode at 60 dB SPL) before and after consumption of coffee (unfilled triangles connected with solid line) and milk (filled triangles with dashed line) for right ear (red colour) and left ear (blue colour).

Figure 3 shows the mean global amplitude of TEOAE (recorded in linear mode at 60 dB SPL) for both ears before and after consumption of coffee and milk. The mean amplitude of TEOAE was larger in the right ear across the conditions ( *i.e.*, before and after consumption of coffee and milk). Further, the mean amplitudes of TEOAE before and after consumption of coffee or milk were similar for both ears. The Shapiro-Wilk test revealed that the amplitude of TEOAE of both ears across conditions was normally distributed. Repeated-measures ANOVA was carried out with ears (right and left), conditions (before and after consumption), and drink (coffee and milk) as repeated measures. Results showed no significant effect of ear [F(1,46)=2.851, p=0.1], condition [F(1,46)=0.604, p=0.441], and drink [F(1,46)=0.288, p=0.594] on the amplitude of TEOAE. Further, no significant interaction was found between ears and conditions [F(1,46)=1.267, p=0.266], drink and conditions [F(1,46)=0.752, p=0.39], ears and drink [F(1,46)=0.959, p=0.333], and ears, drink, and conditions [F(1,46)=0.146, p=0.704]. Bayesian repeated measures ANOVA showed extreme evidence in favour of the null hypothesis for the effect of conditions [BF _10_=0.002] and drink [BF _10_= 0.002] on the amplitude of TEOAEs. Further, it showed anecdotal evidence for the effect of ears [BF _10_=1] on the amplitude of TEOAE which suggests more data need to be collected to draw a firm conclusion (Dienes, 2014).

### Contralateral suppression of transient evoked otoacoustic emissions

**Figure 4.**
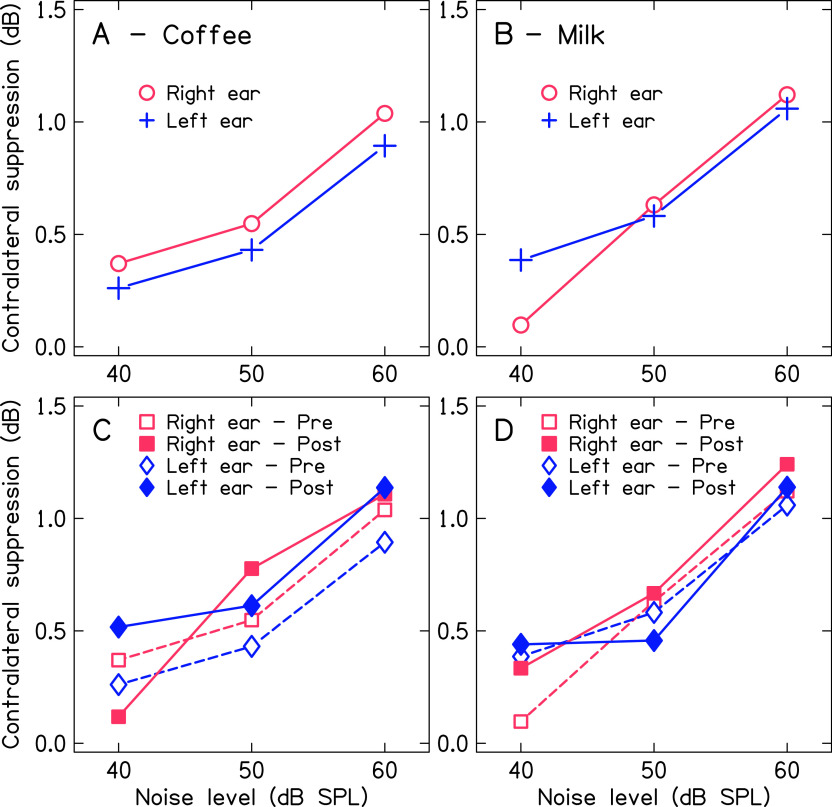
Mean contralateral suppression of TEOAE before and after consumption of coffee and milk and across the levels on noise in the contralateral ear for both ears. Panel A shows the mean contralateral suppression of TEOAE for both ears before consumption of coffee. Panel B shows the mean contralateral suppression of TEOAE for both ears before consumption of milk. Panel C shows the mean contralateral suppression of TEOAE for both ears before and after consumption of coffee. Panel D shows the mean contralateral suppression of TEOAE for both ears before and after consumption of milk.

**Figure 5.**
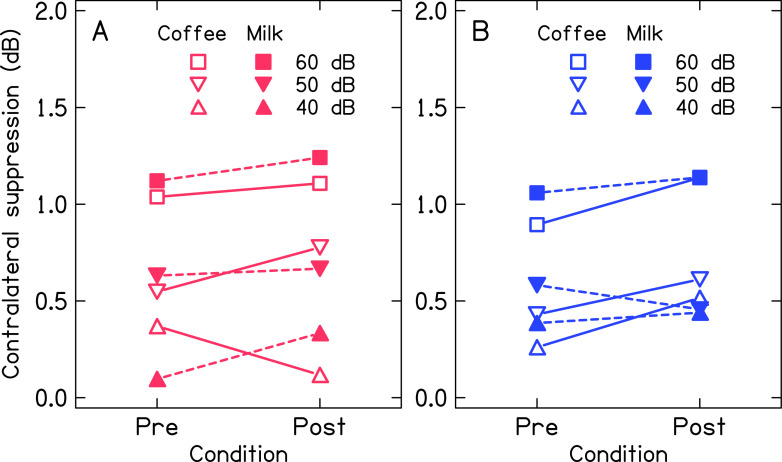
Mean contralateral suppression of TEOAE across levels of noise in the contralateral ear before and after consumption of coffee and milk in right ear (panel A) and left ear (panel B).

Figures 4 and 5 show the mean contralateral suppression of TEOAE for both ears across the levels of noise before and after consumption of coffee and milk. Panel A and panel B of Figure 4 show the mean contralateral suppression of TEOAE across noise levels for baseline measurement. The mean contralateral suppression was slightly lower in the left ear compared to the right ear. Further, the mean contralateral suppression of TEOAE decreased with the reduction in the level of noise in the contralateral ear. Panels C and D of Figure 4 show the mean contralateral suppression of TEOAE before and after consumption of coffee and milk respectively for both ears. The results for the same are depicted in Figure 5 for better visualization. The mean contralateral suppression before and after consumption of milk was similar at each level of noise for both ears. In contrast, a slightly greater suppression was noted after consumption of coffee at each level of noise for both ears, except for the right ear at 40 dB noise.

The Shapiro-Wilk test revealed that the magnitude of contralateral suppression of TEOAE across conditions and levels of noise for both ears were not normally distributed. To investigate the effect of conditions (before and after consumption) and drink (coffee and milk) on the contralateral suppression of TEOAE, the Friedman test was carried out separately for each levels of noise (60 dB, 50 dB, and 40 dB) and ears (right and left). Results showed no significant difference for the magnitude of contralateral suppression of TEOAE before and after consumption of coffee and milk at each level of noise for right ear [60 dB (χ ^2^_(3)_=4.021, p=0.259); 50 dB (χ ^2^_(3)_=3.194, p=0.363); and 40 dB (χ ^2^_(3)_=0.734, p=0.865)] and left ear [60 dB (χ ^2^_(3)_=3.952, p=0.267); 50 dB (χ ^2^_(3)_=1.54, p=0.673); and 40 dB (χ ^2^_(3)_=4.625, p=0.201)]. Further, to investigate the effect of noise level on the contralateral suppression of TEOAE, the data obtained before consumption of coffee was subjected to the Friedman test separately for both ears. Results showed a significant effect of noise level on the contralateral suppression of TEOAE for both ears [right ear (χ ^2^_(2)_=39.542, p<0.001); left ear (χ ^2^_(2)_=39.872, p<0.001)]. Pairwise comparison using Wilcoxon signed ranks test showed the contralateral suppression of TEOAE was significantly different between noise levels 60 dB and 50 dB [right ear (Z=4.723, p<0.001); lef ear (Z=4.482, p<0.001)], 60 dB and 40 dB [right ear (Z=4.723, p<0.001); lef ear (Z=4.54, p<0.001)], and 50 dB and 40 dB [right ear (Z=3.377, p=0.001); left ear (Z=3.549, p<0.001)]. In addition, to investigate the effect of ear on the contralateral suppression of TEOAE, the data obtained before consumption of coffee were subjected to the Wilcoxon signed ranks test. It showed the contralateral suppression of TEOAE was not significantly different between ears at each level of noise [60 dB (Z=1.208, p=0.227); 50 dB (Z=0.688, p=0.492); 40 dB (Z=0.215, p=0.829)].

## Discussion

Results of the present study showed no significant effect of coffee on the amplitude of TEOAE. This finding is consistent with those of Drepath *et al.* ^23^ which showed no effect of coffee on the amplitude of DPOAEs in humans. These findings suggests no effect of caffeine on the amplitude of OAEs in humans. But, in contrast to the findings of human studies, animal studies have reported an effect of caffeine on the amplitude of DPOAEs. Bobbin ^27^ reported a stimulus level-dependent effect of caffeine on the amplitude of DPOAE. The amplitude of DPOAE was found to be reduced when elicited with lower-intensity stimuli and the amplitude was increased when elicited with higher-intensity stimuli. In addition, Bobbin ^27^ also investigated the effect of caffeine on the compound action potentials (CAP), summating potential (SP), cochlear microphonics (CM) and latency of N1. The caffeine had a suppressive effect on the CAP, SP, and latency of N1. Bobbin ^27^ attributed the reduction in the amplitude of DPOAE at low intensity to diminished amplifier function of OHCs in the cochlea, which is a consequence of caffeine. In the cochlea, caffeine causes activation of Ca ^2+^-dependent K ^+^ channels in the OHCs, which leads to hyperpolarization of the OHCs and subsequently suppresses the amplifier function of OHCs. ^24^^–^^26^^,^^30^^,^^31^ Recently, Castellano-Muñoz *et al.* ^32^ investigated the effect of caffeine on the electrical properties of OHCs and postsynaptic activity in auditory fibers. Results showed caffeine had no effect on the electrical properties of OHCs, but it had an effect on the postsynaptic activity in auditory fibers. The findings of the above study suggest that functioning of the OHCs may not be affected by caffeine, and thus the amplitude of OAE could be similar before and after consumption of caffeine.

The present study also investigated the effect of coffee on the contralateral suppression of TEOAE. The contralateral suppression of TEOAE was measured by presenting white noise to the contralateral ear at 40, 50, and 60 dB SPL. Results showed an increase in the magnitude of contralateral suppression of TEOAE with an increase in the level of noise in the contralateral ear. These findings are consistent with results of several investigations. ^33^^–^^36^ The increase in contralateral suppression of TEOAE with noise level has been attributed to the strength of efferent activity. Further, results of the present study showed a slightly greater contralateral suppression after coffee consumption; however, the difference was not significant. As studies investigating the effect of caffeine on the contralateral suppression of TEOAE are not available in the literature, the results of the present study cannot be compared with other investigations. Further, although findings of the present study showed no significant effect of coffee or caffeine on the contralateral suppression of TEOAE, similar studies are essential before generalizing the findings.

Based on the findings of the present study, we understand that consuming coffee before an audiological evaluation has no significant effect on the amplitude of TEOAE and contralateral suppression of TEOAE. However, there are few limitations to the present study. In the literature, studies investigating the effect of caffeine on the ABR have shown a dose-dependent effect of caffeine on the peaks of ABR. ^16^ A similar a dose dependent effect of caffeine could be present on the amplitude of TEOAE and contralateral suppression of TEOAE. However, in the present study a fixed amount of coffee was provided to participants, thus currently it is not understood whether increasing the dose of caffeine would have any effect on the amplitude of TEOAE and contralateral suppression of TEOAEs. Further, the amount of caffeine present in coffee is dependent on the type of coffee ( *i.e.*, brewed, instant, or decaffeinated). ^37^^,^^38^ In the present study instant coffee was given to participants, which contains lower amount of caffeine compared to brewed coffee. Therefore, if coffee has a dose-dependent effects of caffeine on the TEOAE and contralateral suppression of TEOAE, then findings of the present study cannot be generalized to all types of coffee.

To conclude, findings of the present study showed no effect of coffee on the findings of TEOAE. The procedure used for recording the non-linear TEOAE in the present research was similar to the protocol used in clinics for routine evaluation. Thus, based on findings of the present study, we understand that consuming coffee before an audiological evaluation may not have negative effects on the amplitude of TEOAE.

## Data availability

Mendeley Data: The effect of coffee on TEOAE and contralateral suppression of TEOAE, https://doi.org/10.17632/p4pgd57zgd.1 ^39^

This project contains the following underlying data:

- CSTEOAE_linear.csv (contains data of contralateral suppression of TEOAE)
- Read Me.txt (description to understand the variables in data files)
- TEOAE_linear.csv (contains data of amplitude of TEOAE recorded in linear mode in baseline and contralateral conditions)
- TEOAE_non-linear.csv (contains data of amplitude of TEOAE recorded in non-linear mode)

Data are available under the terms of the Creative Commons Attribution 4.0 International license (CC-BY 4.0).
