# Supplementary material for: The effect of coffee on contralateral suppression of transient evoked otoacoustic emissions
Source: F1000Res. 2023 Sep 13;11:878. Originally published 2022 Aug 2. [Version 2] doi: 10.12688/f1000research.122851.2 (PMC10568215; doi:10.12688/f1000research.122851.2)
Supplement: Peer review history

# Reviewer report: Reviewer response for version 2

**DOI:** 10.5256/f1000research.155503.r206293

**Published:** 2023-11-09

**Authors:** Wiktor Jedrzejczak

**Affiliations:** 1 World Hearing Center, Institute of Physiology and Pathology of Hearing, Warsaw, Poland

I appreciate all the corrections. I have some further suggestions for improvement of the manuscript.

 There is very limited number of references to OAE studies. All cited OAE studies except one are older than 20 years. There is a plenty of recent studies to which the authors could refer to. On the other hand when introducing OAEs I suggest to reference at least one paper by David Kemp who discovered them.

 When citing a book (reference number 8) please cite actual pages to which you refer or alternatively please cite the papers which are related to this area.

 Please try to more clearly formulate the aim of the study. The sentence “Therefore, there is a need to understand the effect of caffeine on the efferent activity.” Is rather vague.

 Indeed, there are no studies on suppression of OAE in relation to coffee, but there are studies showing what are the fluctuations of suppression or what are the differences in suppression across measurements at the same day. There are even studies done on the same type of equipment. Please try to compare present results to these studies.

Is the work clearly and accurately presented and does it cite the current literature?

Partly

If applicable, is the statistical analysis and its interpretation appropriate?

Partly

Are all the source data underlying the results available to ensure full reproducibility?

Yes

Is the study design appropriate and is the work technically sound?

Partly

Are the conclusions drawn adequately supported by the results?

Partly

Are sufficient details of methods and analysis provided to allow replication by others?

Partly

Reviewer Expertise:

Neuroscience, audiology, signal processing, otoacoustic emissions

I confirm that I have read this submission and believe that I have an appropriate level of expertise to confirm that it is of an acceptable scientific standard, however I have significant reservations, as outlined above.

# Reviewer report: Reviewer response for version 2

**DOI:** 10.5256/f1000research.155503.r206294

**Published:** 2023-10-11

**Authors:** Sandeep Maruthy

**Affiliations:** 1 Department of Audiology, All India Institute of Speech and Hearing, Mysuru, Karnataka, India

I can find that the revised manuscript is in a much better shape with authors addressing all the concerns raised. The manuscript can be accepted for indexing, provided the authors address some minor concerns listed below:

 1) In the page 8, the authors state, 'As studies investigating the effect of caffeine on the contralateral suppression of TEOAE are not available in the literature', which is not true. They themselves have quoted Drepath et al. at many instances in the article. I suggest rephrasing.

 2) The authors in the Introduction section claim that their study will help in generalizing the results of Drepath et al., who did not find significant difference of coffee consumption on CSOAEs. The authors in this study found results similar to Drepath et al. Yet, they conclude that 'similar studies are essential before generalizing the results' (same statement repeated). This surprises me. When two studies at different points of time, by different groups of researchers have shown the same results, what is the reason for authors to call for another such study? How is that expected to be different? In my opinion it is not required. I suggest that authors discuss it differently.

 3) The findings support the absence of effect for dosage used in this study (1.3g coffee powder mixed with two spoons of powdered milk & 150 mL water). During the discussion of their results, the authors, rightly so, speculate dose dependent effect of caffeine. I suggest the authors to state clearly that dosage of caffeine used in this study or any lower than this will not lead to significant effects on CSOAEs. However, the effect of caffeine dosage higher than that used in the study needs to be explored in future.

Is the work clearly and accurately presented and does it cite the current literature?

Partly

If applicable, is the statistical analysis and its interpretation appropriate?

Partly

Are all the source data underlying the results available to ensure full reproducibility?

Yes

Is the study design appropriate and is the work technically sound?

Yes

Are the conclusions drawn adequately supported by the results?

Yes

Are sufficient details of methods and analysis provided to allow replication by others?

Yes

Reviewer Expertise:

Audiology, electrophysiology

I confirm that I have read this submission and believe that I have an appropriate level of expertise to confirm that it is of an acceptable scientific standard.

# Reviewer report: Reviewer response for version 2

**DOI:** 10.5256/f1000research.155503.r206292

**Published:** 2023-10-06

**Authors:** Chhayakant Patro

**Affiliations:** 1 Department of Speech-Language Pathology & Audiology, Towson University, Towson, MD, USA

I am delighted to accept this paper for publication as the authors have comprehensively addressed all the concerns raised during the last peer review process.

Is the work clearly and accurately presented and does it cite the current literature?

Partly

If applicable, is the statistical analysis and its interpretation appropriate?

Yes

Are all the source data underlying the results available to ensure full reproducibility?

Yes

Is the study design appropriate and is the work technically sound?

Yes

Are the conclusions drawn adequately supported by the results?

Partly

Are sufficient details of methods and analysis provided to allow replication by others?

Partly

Reviewer Expertise:

Peripheral physiology, speech perception, and aging

I confirm that I have read this submission and believe that I have an appropriate level of expertise to confirm that it is of an acceptable scientific standard.

# Reviewer report: Reviewer response for version 1

**DOI:** 10.5256/f1000research.134894.r146365

**Published:** 2022-12-13

**Authors:** Sandeep Maruthy

**Affiliations:** 1 Department of Audiology, All India Institute of Speech and Hearing, Mysuru, Karnataka, India

The study compared the amplitude of OAEs and the magnitude of contralateral suppression of OAEs before and after consuming coffee. The results show no significant difference between the two conditions, which the authors interpret as ‘ *no effect of coffee on OAEs and contralateral suppression of OAEs.*’ The authors have used a robust research design, an acceptable sample size and have made reasonable attempts to control the possible extraneous variables. Overall, I appreciate the authors for their work. It is a work worth indexing and of interest to the potential readers of this journal. However, the manuscript needs a significant revision before I can recommend this for indexing. Following are my section-wise comments (a few of them in the form of questions to the authors) which I suggest the authors consider while revising the manuscript.

**Title**

 The title reflects only the contralateral suppression of TEOAEs, and misses out on reflecting the amplitude of OAEs.

**Abstract**

 The background should highlight the need for the study. Just stating that studies have rarely investigated its effect on OAEs may not be sufficient to justify the need for the current study.

 I suggest the authors to confine the reporting to the statistical test findings and not report the mean difference in the abstract.

 The abstract may have to be re-written after considering the comments of the other sections.

**Introduction**

- The authors must explain in more detail the influence of caffeine on the biochemistry due to which they expect OAEs and contralateral suppression of OAEs (CSOAEs) to change. The last sentence of the first paragraph mentions it in brief. The authors can elaborate it in the context of OAEs and CSOAEs. Also, later in the Discussion section, the authors have explained it based on Castellano-Muñoz et al. and Bobbin’s studies, which needs mention in introduction.
- The word ‘ *Further*’ is not necessary in a couple of instances in the first two paragraphs and I suggest removing it.
- The transition of thought from the second to the third paragraph could be smoother. I suggest the authors make the necessary change.
- The abbreviation ‘OAEs’ once used, needs to be continued. For example, second line of the fourth paragraph has the full form.
- In the second line of the fifth paragraph, the sentence should read as ‘ *caffeine on the cochlea have reported that’* instead of ‘ *caffeine on the cochlea have reported.*’
- What is a positive effect? Needs clarity.
- Earlier studies have shown improvement in SPIN with caffeine intake; changes in SPIN are associated with changes in CSOAES; therefore, the authors expected caffeine intake to change the CSOAEs. However, the justification is not clearly narrated in the manuscript.
- Drepath et al. reported no significant effect of coffee on the amplitude of DPOAEs, but Bobbin found a significant effect. The presence of equivocal findings among previous studies alone cannot justify taking up a new study. I suggest that the authors critically analyze the method used in these studies and identify the reason for differences in the findings, to justify the study and the to support the method used here.
- The earlier studies have used DPOAEs while in the current study the authors used TEOAEs. Why did the authors prefer TEOAEs over DPOAEs? Considering that the physiological mechanisms of these two types of OAEs are different, can we not expect different findings in the two? If so, is it reasonable to compare the current findings with that of the previous studies where a different type of OAE was used?
- The statement ‘ *This improved transmission in the afferent pathways could also elicit stronger activity in the efferent pathway*’ needs to be supported with a citation.
- In the statement ‘ *However, none of the studies have investigated the effect of caffeine of the efferent activity*’, I think the authors are referring to ‘efferent auditory activity’. Please specify.
- Many studies have shown an association between CSOAEs and SPIN. All of them need to be cited in the manuscript. For example, Maruthy et al. (2017). Also, not all the studies that probed this relationship have shown association between the two. Therefore, I prefer that the authors mention ‘ *some of the studies that probed ………….’*

**Method**

- How was the sample size determined? Give details.
- The statement ‘ *The acoustic reflex threshold for white noise was greater than 70 dB SPL for all the participants*’ can be moved to earlier in the paragraph
- The first sentence in the procedure is a repetition of what is in the previous paragraph. Remove it from the procedure.
- ‘ *Fitted to the ear test ear*’ and ‘ *fitted to the contralateral ear*’ can be changed to ‘inserted’ 
- What was the need to record contralateral suppression at 3 different noise levels? Did the authors expect differential effects of coffee across suppressor intensities? This needs a strong justification in the manuscript. Otherwise, I suggest that the authors present results of either 50dBSPL or 60dBSPL alone. If they wish to retain it as it is, intensity as a variable needs a mention and justification in the Introduction section.
- I have a similar comment for recording the effect in both ears of the subjects. Did the authors expect different effects in the two ears? If so, it needs to be justified.
- I suggest that the authors explain the significance of recording OAEs in both linear and nonlinear paradigms. Considering that contralateral suppression of OAEs is higher in the linear paradigm, the authors could have restricted it to only the linear paradigm.
- It is stated that ‘ *All the recordings of TEOAE were obtained without disturbing the placement of OAE probe (i.e., single-fit condition)*’. The authors need to specify what measures they took to ensure that probe did not move while drinking coffee or milk, and in the next 1 hour until the second recording of OAEs was made.
- As I understood, the authors recorded CSOAEs after the baseline TEOAEs. I do not see a mention of that either in the text or in the Figure 1. Please make the necessary change.
- Mention the rationale for 1-hour interval after coffee consumption.
- The statement ‘ *The TEOAEs obtained in the second session were similar to the first session and were referred as ‘follow-up measurements*’. Do authors mean that the procedure was the same?
- Rather than calling them ‘ *baseline*’ and ‘ *follow up*’ measurements, I suggest ‘pre-drink’ and ‘post-drink’ measurements. This I think these terms will also compliment with the term given for this variable (drink) in the results section.
- Coffee preparation is one of the methods used in India. For example, coffee in USA is typically not prepared with milk or milk powder. I suggest the authors to mention that this is a method of coffee preparation followed in India.
- Please explain the rationale for using 1.3g of coffee. How does it relate to the coffee dosage used in the earlier studies?
- As mentioned earlier, Figure 1 does not reflect CSOAE measurements.
- Provide supporting reference to the 6 dB and 3dB criteria used.
- The presence of 6dB in nonlinear mode and 3 dB in linear mode should have been a participant selection criterion.
- I suggest that the authors mention the target response measures in the method section.

**Results**

- The authors should talk about the normality test upfront and then describe the trend in the mean amplitude.
- ‘ *conditions*’ to be changed to ‘condition’
- In Figure 2, I believe the unit is dB SPL. Please specify.
- Comparing between ears and across intensities of suppressors needs to be stated in the objectives. Otherwise, I suggest the authors to remove it from the results section.
- In the results of CSOAEs, the normality test revealed non-normal distribution of the data. Accordingly, Friedman test is used. In such a case, presenting median and IQR is more meaningful than mean and SD.
- ‘ *both ears*’ to be changed to ‘in the two ears’ in several places. 
- Error bars or the appropriate measure of variance is not reflected in Figure 4 and 5. Please include. I suggest to show median and IQR.
- The Y-axis in Figure 4 and 5 should read as ‘magnitude of CSOAEs (dB)’.
- The information in Figures 4 and 5 are the same but represented differently. I suggest retaining Figure 5 and removing Figure 4. 

**Discussion **

- The statement ‘ *The findings of the above study suggest that functioning of the OHCs may not be affected by caffeine, and thus the amplitude of OAE could be similar before and after consumption of caffeine’* is too profound for what authors can derive from their study. It should be read in the context of the dosage of coffee used in this study. I suggest that the authors tone down the inference drawn.
- This study showed the absence of significant effect of coffee on CSOAEs in presence of improvement in SPIN (as shown by earlier studies). Does it reflect that improvements in SPIN observed by them is not efferent mediated? The authors can consider discussing this direction.
- The results showed some effect, but that was not significant. Therefore, instead of concluding it as ‘ *findings of the present study showed no effect of coffee on the findings of TEOAE’ the authors can phrase it as ‘findings suggest no effect of coffee*’.
- The authors in the last statement of the manuscript state ‘ *may not have negative effects on the amplitude of TEOAE,*’ but earlier in the manuscript, they expected positive effects. This is contradictory. I believe they meant that clinical measurement of TEOAEs and the inferences drawn from it don’t change with coffee consumption. Please make changes in the statement accordingly. 
- To summarize, one may find more criticisms in my peer-review report than appreciations. This does not take away the credit for the good work done by the authors and the significant contribution of their work to science. I suggest that they revise it based on the inputs provided and submit the revised manuscript for reconsideration. 

Is the work clearly and accurately presented and does it cite the current literature?

Partly

If applicable, is the statistical analysis and its interpretation appropriate?

Partly

Are all the source data underlying the results available to ensure full reproducibility?

Yes

Is the study design appropriate and is the work technically sound?

Yes

Are the conclusions drawn adequately supported by the results?

Yes

Are sufficient details of methods and analysis provided to allow replication by others?

Yes

Reviewer Expertise:

Audiology, electrophysiology

I confirm that I have read this submission and believe that I have an appropriate level of expertise to confirm that it is of an acceptable scientific standard, however I have significant reservations, as outlined above.

# Response

**Published:** 2023-09-01

**Authors:** Mohan Kalaiah

**Affiliations:** Kasturba Medical College, Mangalore, India

We thank you for peer reviewing the manuscript.

 Based on your recommendations, manuscript has been modified. See details below:

**Title**

**Reviewer comment: **The title reflects only the contralateral suppression of TEOAEs, and misses out on reflecting the amplitude of OAEs.

**Authors reply:** Title changed to “The effect of coffee on transient evoked otoacoustic emissions and contralateral suppression of transient evoked otoacoustic emissions”

**Abstract**

**Reviewer comment:** The background should highlight the need for the study. Just stating that studies have rarely investigated its effect on OAEs may not be sufficient to justify the need for the current study.

 I suggest the authors to confine the reporting to the statistical test findings and not report the mean difference in the abstract.

 The abstract may have to be re-written after considering the comments of the other sections.

**Authors reply: **Statement on mean difference was removed from the abstract. Abstract modified

**Introduction**

**Reviewer comment:** The authors must explain in more detail the influence of caffeine on the biochemistry due to which they expect OAEs and contralateral suppression of OAEs (CSOAEs) to change. The last sentence of the first paragraph mentions it in brief. The authors can elaborate it in the context of OAEs and CSOAEs. Also, later in the Discussion section, the authors have explained it based on Castellano-Muñoz et al. and Bobbin’s studies, which needs mention in introduction.

**Authors reply: **We agree with the recommendation 

**Reviewer comment: **The word ‘ *Further*’ is not necessary in a couple of instances in the first two paragraphs and I suggest removing it.

**Authors reply:** removed 

**Reviewer comment: **The transition of thought from the second to the third paragraph could be smoother. I suggest the authors make the necessary change.

**Authors reply: **modified 

**Reviewer comment: **The abbreviation ‘OAEs’ once used, needs to be continued. For example, second line of the fourth paragraph has the full form.

**Authors reply:** modified 

**Reviewer comment: **In the second line of the fifth paragraph, the sentence should read as ‘ *caffeine on the cochlea have reported that’* instead of ‘ *caffeine on the cochlea have reported.*’

**Authors reply: **modified 

**Reviewer comment:** What is a positive effect? Needs clarity.

**Authors reply:** Positive effect refer to improved neural transmission. Same has been added to the manuscript.  

**Reviewer comment: **The earlier studies have used DPOAEs while in the current study the authors used TEOAEs. Why did the authors prefer TEOAEs over DPOAEs? Considering that the physiological mechanisms of these two types of OAEs are different, can we not expect different findings in the two? If so, is it reasonable to compare the current findings with that of the previous studies where a different type of OAE was used?

**Authors reply: **Yes, we agree that different findings are possible. TEOAEs were used in the present study as studies measuring the TEOAEs are not available in literature.  

**Reviewer comment: **The statement ‘ *This improved transmission in the afferent pathways could also elicit stronger activity in the efferent pathway*’ needs to be supported with a citation.

**Authors reply:** Statement modified to ‘ *This improved transmission in the afferent pathways could have an influence on the activity in the efferent pathway*’ 

**Reviewer comment: **In the statement ‘ *However, none of the studies have investigated the effect of caffeine of the efferent activity*’, I think the authors are referring to ‘efferent auditory activity’. Please specify.

**Authors reply:** modified. ‘efferent activity’ changed to ‘efferent auditory activity’ 

**Reviewer comment:** Many studies have shown an association between CSOAEs and SPIN. All of them need to be cited in the manuscript. For example, Maruthy et al. (2017). Also, not all the studies that probed this relationship have shown association between the two. Therefore, I prefer that the authors mention ‘ *some of the studies that probed ………….’*

**Authors reply**: references added.  

**Method**

**Reviewer comment:** The statement ‘ *The acoustic reflex threshold for white noise was greater than 70 dB SPL for all the participants*’ can be moved to earlier in the paragraph

**Authors reply:** done 

**Reviewer comment: **The first sentence in the procedure is a repetition of what is in the previous paragraph. Remove it from the procedure.

**Authors reply:** removed 

**Reviewer comment: **‘ *Fitted to the ear test ear*’ and ‘ *fitted to the contralateral ear*’ can be changed to ‘inserted’

**Authors reply:** done 

**Reviewer comment:** What was the need to record contralateral suppression at 3 different noise levels? Did the authors expect differential effects of coffee across suppressor intensities? This needs a strong justification in the manuscript. Otherwise, I suggest that the authors present results of either 50dBSPL or 60dBSPL alone. If they wish to retain it as it is, intensity as a variable needs a mention and justification in the Introduction section.

**Authors reply:** As the paper is already published we decided not to remove the data from the manuscript. However, for the purpose of statistical analysis only 60 dB noise was considered.  

**Reviewer comment:** I have a similar comment for recording the effect in both ears of the subjects. Did the authors expect different effects in the two ears? If so, it needs to be justified.

**Authors reply: **Many studies have documented a higher contralateral suppression of OAE in the right, suggesting an asymmetry in the magnitude of suppression between ears. Thus, in the present investigation the effect of caffeine on the contralateral suppression of TEOAE was measured in both ears.  

**Reviewer comment: **I suggest that the authors explain the significance of recording OAEs in both linear and nonlinear paradigms. Considering that contralateral suppression of OAEs is higher in the linear paradigm, the authors could have restricted it to only the linear paradigm.

**Authors reply:** Non-linear TEOAEs are commonly recorded for clinical applications. While linear TEOAEs are commonly used during measurement of contralateral suppression of TEOAE. As recommended, in the present study, the contralateral suppression of TEOAE was measured using linear TEOAE only.  

**Reviewer comment:** It is stated that ‘ *All the recordings of TEOAE were obtained without disturbing the placement of OAE probe (i.e., single-fit condition)*’. The authors need to specify what measures they took to ensure that probe did not move while drinking coffee or milk, and in the next 1 hour until the second recording of OAEs was made.

**Authors reply:** All the recordings in one session was obtained in single-fit condition. Two sessions were present in one day with a gap of 1 hour (before and after).  

**Reviewer comment:** As I understood, the authors recorded CSOAEs after the baseline TEOAEs. I do not see a mention of that either in the text or in the Figure 1. Please make the necessary change.

**Authors reply:** Yes, initially ‘baseline’ TEOAE was recorded and after that the TEOAE was recorded by delivering noise to the contralateral ear.  

**Reviewer comment: **Mention the rationale for 1-hour interval after coffee consumption.

**Authors reply: **The effects from caffeine reach peak levels after 30 to 45 minutes of consumption. Thus, 1-hour interval was used between sessions.  

**Reviewer comment: **The statement ‘ *The TEOAEs obtained in the second session were similar to the first session and were referred as ‘follow-up measurements*’. Do authors mean that the procedure was the same?

**Authors reply: **Yes 

**Reviewer comment: **Rather than calling them ‘ *baseline*’ and ‘ *follow up*’ measurements, I suggest ‘pre-drink’ and ‘post-drink’ measurements. This I think these terms will also compliment with the term given for this variable (drink) in the results section.

**Authors reply: **done 

**Reviewer comment: **Coffee preparation is one of the methods used in India. For example, coffee in USA is typically not prepared with milk or milk powder. I suggest the authors to mention that this is a method of coffee preparation followed in India.

**Authors reply: **Agreed and added to manuscript

**Reviewer comment: **Please explain the rationale for using 1.3g of coffee. How does it relate to the coffee dosage used in the earlier studies?

**Authors reply: **1.3 g was the amount of coffee available in one sachet. It is typical serving size for one cup of coffee. 

**Reviewer comment: **As mentioned earlier, Figure 1 does not reflect CSOAE measurements.

**Authors reply: **Details added to the legend of figure.

**Reviewer comment: **The presence of 6dB in nonlinear mode and 3 dB in linear mode should have been a participant selection criterion.

**Authors reply:** Agreed. We used 6 dB SNR in nonlinear mode as one of the criterion for participant selection in the present study. In linear mode, when the SNR was less than 6 dB OAE was considered as absent.

**Reviewer comment:** I suggest that the authors mention the target response measures in the method section.

**Authors reply: **We measured amplitude of TEOAE in non-linear and linear modes and magnitude of contralateral suppression of TEOAE. Same is mentioned in data analysis section.

**Results**

**Reviewer comment: **The authors should talk about the normality test upfront and then describe the trend in the mean amplitude.

**Authors reply: **Results of normality tests are provided in the manuscript.  

**Reviewer comment: **‘ *conditions*’ to be changed to ‘condition’

**Authors reply: **done 

**Reviewer comment:** In Figure 2, I believe the unit is dB SPL. Please specify.

**Authors reply:** SPL added 

**Reviewer comment:** Comparing between ears and across intensities of suppressors needs to be stated in the objectives. Otherwise, I suggest the authors to remove it from the results section.

**Authors reply: **We prefer not to add the comparison across level of noise as an objective.  

**Reviewer comment: **‘ *both ears*’ to be changed to ‘in the two ears’ in several places.

**Authors reply: **done

**Reviewer comment: **The Y-axis in Figure 4 and 5 should read as ‘magnitude of CSOAEs (dB)’.

**Authors reply: **Axis changed 

**Reviewer comment: **The information in Figures 4 and 5 are the same but represented differently. I suggest retaining Figure 5 and removing Figure 4. 

**Authors reply: **As manuscript is already published we prefer to retain both the figures.

**Discussion**

**Reviewer comment: **The statement ‘ *The findings of the above study suggest that functioning of the OHCs may not be affected by caffeine, and thus the amplitude of OAE could be similar before and after consumption of caffeine’* is too profound for what authors can derive from their study. It should be read in the context of the dosage of coffee used in this study. I suggest that the authors tone down the inference drawn.

**Authors reply: **Modified

**Reviewer comment:** This study showed the absence of significant effect of coffee on CSOAEs in presence of improvement in SPIN (as shown by earlier studies). Does it reflect that improvements in SPIN observed by them is not efferent mediated? The authors can consider discussing this direction.

**Authors reply: **Could be mediated by efferent activity. Findings of Castellano-Muñoz *et al.* has revealed that caffeine resulted in changes in post-synaptic activity in auditory fibers in the absence of changes in OHCs. Thus, involvement of efferent activity may not be ruled out.

**Reviewer comment:** The results showed some effect, but that was not significant. Therefore, instead of concluding it as ‘ *findings of the present study showed no effect of coffee on the findings of TEOAE’ the authors can phrase it as ‘findings suggest no effect of coffee*’.

**Author reply:** modified

**Reviewer comment: **The authors in the last statement of the manuscript state ‘ *may not have negative effects on the amplitude of TEOAE,*’ but earlier in the manuscript, they expected positive effects. This is contradictory. I believe they meant that clinical measurement of TEOAEs and the inferences drawn from it don’t change with coffee consumption. Please make changes in the statement accordingly.

**Author reply:** Agreed 

**Reviewer comment: **To summarize, one may find more criticisms in my peer-review report than appreciations. This does not take away the credit for the good work done by the authors and the significant contribution of their work to science. I suggest that they revise it based on the inputs provided and submit the revised manuscript for reconsideration.

**Author reply: **Thank you for agreeing to review the manuscript and providing recommendations.

# Reviewer report: Reviewer response for version 1

**DOI:** 10.5256/f1000research.134894.r153018

**Published:** 2022-12-01

**Authors:** Chhayakant Patro

**Affiliations:** 1 Department of Speech-Language Pathology & Audiology, Towson University, Towson, MD, USA

This manuscript reports the results of an experiment that investigated the effects of caffeine intake on transient evoked otoacoustic emissions (TEOAEs) and contralateral suppression of TEOAEs, in a group of young listeners with normal hearing (N = 52). The results indicated that caffeine intake had no effects on absolute TEOAE amplitudes and had negligible effects on the contralateral suppression of TEOAEs. The following sections of general comments summarize my main comments and concerns. Specific comments (e.g., line-by-line editing) are not included as the line numbers are not available (to me).

- The use of cross-over design is innovative. Some individuals may have a greater sensitivity to the effects of caffeine than others and by treating each participant as his/her own control, such individual differences were accounted for.
- Any general comments regarding the effects of caffeine on auditory/ speech perception could be removed from the introduction section since the experiment focused on physiological effects and not on the perceptual consequences of caffeine consumption.
- Some sections of the manuscript are questionable because of a lack of coherence, and under-developed arguments. For example, the authors should give a clearer motivation for the choice of stimuli and conditions. Why did the authors hypothesize that TEOAEs could be more sensitive to the effects of caffeine than the DPOAEs (since the DPOAEs have produced null results, as the authors rightly acknowledge)?
- Throughout the paper (including the discussion section), comparisons to DPOAEs should be limited.
- “ *Further, studies investigating the effect of caffeine on the speech perception have reported a positive effect of caffeine on the perception of speech in noise.*” – citations are needed here. Were the “positive effects” mediated by improved efferent activity? If not, those results are not relevant here.
- Although the authors cite some of the classic studies on OAEs, more recent citations are needed.
- Precisely how much caffeine was provided to the study participants?
- Were the acoustic reflexes present at all the test frequencies?
- Did the authors conduct an interview/ provide a questionnaire to determine whether the participants had a history of otological problems? Did the authors quantify the amount of noise exposure? This information is relevant because prolonged/repetitive exposure to loud sounds may lead to (subclinical) cochlear dysfunction and may obscure the observation of any potential relationship between caffeine consumption and cochlear function. At least, this needs to be addressed in the discussion section.
- Involving a large group of participants is a major strength.
- “ *Further, the TEOAEs recorded in the linear mode were considered to be present if the global SNR was at least 3 dB SNR*.” Citation(s) needed here. Was the +3 dB SNR criterion based on previous studies?
- Dienes, 2014: numeric reference formatting need.
- The statistical analyses did not reveal significant effects of caffeine consumption on contralateral suppression of TEOAEs. Still, the authors claim that “ *TEOAE was slightly larger after consumption of coffee in both ears*” in the abstract, based on their qualitative judgment. Their observations may be highlighted in the discussion section, not in the abstract.
- What do the error bars in figures 2 and 3 depict?
- Please provide error bars in figures 4 and 5.

Is the work clearly and accurately presented and does it cite the current literature?

Partly

If applicable, is the statistical analysis and its interpretation appropriate?

Yes

Are all the source data underlying the results available to ensure full reproducibility?

Yes

Is the study design appropriate and is the work technically sound?

Yes

Are the conclusions drawn adequately supported by the results?

Partly

Are sufficient details of methods and analysis provided to allow replication by others?

Partly

Reviewer Expertise:

Peripheral physiology, speech perception, and aging

I confirm that I have read this submission and believe that I have an appropriate level of expertise to confirm that it is of an acceptable scientific standard, however I have significant reservations, as outlined above.

# Response

**Published:** 2023-09-01

**Authors:** Mohan Kalaiah

**Affiliations:** Kasturba Medical College, Mangalore, India

We thank you for peer reviewing the manuscript.

 Based on your recommendation manuscript has been modified. See details below:

**Reviewer comment: **Any general comments regarding the effects of caffeine on auditory/ speech perception could be removed from the introduction section since the experiment focused on physiological effects and not on the perceptual consequences of caffeine consumption.

**Authors reply: **Agree. But no changes made to the manuscript.

**Reviewer comment: **Some sections of the manuscript are questionable because of a lack of coherence, and under-developed arguments. For example, the authors should give a clearer motivation for the choice of stimuli and conditions. Why did the authors hypothesize that TEOAEs could be more sensitive to the effects of caffeine than the DPOAEs (since the DPOAEs have produced null results, as the authors rightly acknowledge)?

**Authors reply: **Agree. We aimed to investigate the effect of coffee on the TEOAE.

**Reviewer comment: **Throughout the paper (including the discussion section), comparisons to DPOAEs should be limited.

**Authors reply: **DPOAE results are included as TEOAE results are not available in literature.

**Reviewer comment**: “ *Further, studies investigating the effect of caffeine on the speech perception have reported a positive effect of caffeine on the perception of speech in noise.*” – citations are needed here. Were the “positive effects” mediated by improved efferent activity? If not, those results are not relevant here.

**Authors reply:** Studies have been cited. We do not know if positive effects are mediated by efferent activity.  

**Reviewer comment: **Although the authors cite some of the classic studies on OAEs, more recent citations are needed.

**Authors reply: **References added

**Reviewer comment:** Precisely how much caffeine was provided to the study participants?

**Authors reply:** The amount of caffeine ranged from 27 to 40 mg per cup of coffee. Same information is added to the manuscript.  

**Reviewer comment: **Were the acoustic reflexes present at all the test frequencies?

**Authors reply: **The acoustic reflex threshold was measured for puretones of frequency 500 Hz, 1000 Hz, 2000 Hz, and 4000 Hz and white noise.

**Reviewer comment:** Did the authors conduct an interview/ provide a questionnaire to determine whether the participants had a history of otological problems? Did the authors quantify the amount of noise exposure? This information is relevant because prolonged/repetitive exposure to loud sounds may lead to (subclinical) cochlear dysfunction and may obscure the observation of any potential relationship between caffeine consumption and cochlear function. At least, this needs to be addressed in the discussion section.

**Authors reply: **Yes, details were conducted through interview.  

**Reviewer comment: **Involving a large group of participants is a major strength.

**Reviewer comment:** “ *Further, the TEOAEs recorded in the linear mode were considered to be present if the global SNR was at least 3 dB SNR*.” Citation(s) needed here. Was the +3 dB SNR criterion based on previous studies?

**Authors reply: **Earlier studies measuring contralateral suppression of OAE have used 3 dB SNR criterion. But, recent literature recommends 6 dB SNR or higher SNR for greater reliability. The data was reanalyzed with 6 dB SNR criterion and results are reported.  

**Reviewer comment: **Dienes, 2014: numeric reference formatting need.

**Authors reply: **done 

**Reviewer comment: **The statistical analyses did not reveal significant effects of caffeine consumption on contralateral suppression of TEOAEs. Still, the authors claim that “ *TEOAE was slightly larger after consumption of coffee in both ears*” in the abstract, based on their qualitative judgment. Their observations may be highlighted in the discussion section, not in the abstract.

**Authors reply: **Abstract modified 

**Reviewer comment: **What do the error bars in figures 2 and 3 depict?

**Authors reply: **error bars depict standard deviation. Added to description 

**Reviewer comment: **Please provide error bars in figures 4 and 5.

**Authors reply:** Error bars (SD) added to figure 4. Data showed in figure 5 is same as figure 4.

# Reviewer report: Reviewer response for version 1

**DOI:** 10.5256/f1000research.134894.r147805

**Published:** 2022-09-09

**Authors:** Wiktor Jedrzejczak

**Affiliations:** 1 World Hearing Center, Institute of Physiology and Pathology of Hearing, Warsaw, Poland

**General comments**:

 The manuscript is related to effects of coffee on contralateral suppression of transient evoked otoacoustic emissions (OAEs). The strengths of the manuscript are large group of subjects and the design with repeated measurements. However there are also several weaknesses like lack of information of the caffeine content used for the experiments and lack of any other verification of caffeine influence on the subjects besides OAEs, no comparison with previous studies, old and wrong references to literature, very small references to OAE studies. My more specific comments are attached below.

**Specific comments:**

**Introduction**:

 Several sentences in the introduction should be backed up by references, e.g.:

- “ *In the cochlea, the OAEs are produced spontaneously and also in response to an external acoustic stimuli, referred as spontaneous OAEs and evoked OAEs respectively.”*
- *“The OAEs elicited in response to pure-tones are known as distortion product OAEs (DPOAEs) and stimulus frequency OAEs (SFOAEs).”*
- *“The suppression of OAE obtained by presenting noise to the non-test ear is known as the contralateral suppression of OAE.*”

The authors refer to review by Guinan, but he uses term of inhibition not suppression and it is directly stated why in the referenced paper. This should be at least commented.

**Method:**

- How much caffeine was in the coffee? Please provide information of the amount of caffeine used for the experiments.
- It would be good if the authors evaluated effect of coffee by some other test, to show that coffee influenced the subject at all, e.g. in studies of effect of attention on OAEs the attention level is evaluated by counting of stimuli or by evoked potentials, and then OAE analyses are made.
- How the authors evaluated the middle ear muscle reflex (MEMR)? Several studies point to MEMR interaction with experiments based on contralateral suppression of OAEs. The authors should check the MEMR influence or at least mention lack of it as the limitation of the study.
- Why are OAE parameters calculated by some program, and not taken from the ILO system?
- Provide some rationale for using 3 dB SNR criterion while studies of reliability of OAE suppression recommend using at least 6 dB or even more.

**Results:**

- Please provide error bars on figures 4 and 5.
- Maybe the authors could select smaller dataset with OAEs of SNR > 6dB?

**Discussion:**

- Study is based on TEOAEs so the comparisons to DPOAEs should be limited.
- Please compare results with other studies of suppression on same equipment, especially recent studies of reliability/variability. Even if there are no studies of coffee on suppression of OAEs, there are some studies of reliability of TEOAEs measured by the same equipment. These are very good basis for comparison, and for reference what change in TEOAE suppression would show the significant effect.

**References:**

- Please add references of some OAE review studies.
- Most references are old. Please add references for papers from last 5 years.
- There is in general too small number of references to OAE studies. There is plenty of OAE studies made on the same system with which the results could be compared.
- Mistakes – references to papers in which there is no referenced information. For example: “ *Further, the OAEs elicited in response to short-duration stimuli such as clicks and tone-bursts are known as transient evoked OAEs (TEOAEs).*” which cites Kemp 1978. At this time Kemp did not used the name of TEOAEs.

Is the work clearly and accurately presented and does it cite the current literature?

Partly

If applicable, is the statistical analysis and its interpretation appropriate?

Partly

Are all the source data underlying the results available to ensure full reproducibility?

Yes

Is the study design appropriate and is the work technically sound?

Partly

Are the conclusions drawn adequately supported by the results?

Partly

Are sufficient details of methods and analysis provided to allow replication by others?

Partly

Reviewer Expertise:

neuroscience, audiology, signal processing, otoacoustic emissions

I confirm that I have read this submission and believe that I have an appropriate level of expertise to state that I do not consider it to be of an acceptable scientific standard, for reasons outlined above.

# Response

**Published:** 2023-09-01

**Authors:** Mohan Kalaiah

**Affiliations:** Kasturba Medical College, Mangalore, India

We thank you for peer reviewing the manuscript.

 Based on your recommendations, manuscript has been modified. See details below:

 Introduction:

**Reviewer comment:** Several sentences in the introduction should be backed up by references, e.g.:

 “In the cochlea, the OAEs are produced spontaneously and also in response to an external acoustic stimuli, referred as spontaneous OAEs and evoked OAEs respectively.”

 “The OAEs elicited in response to pure-tones are known as distortion product OAEs (DPOAEs) and stimulus frequency OAEs (SFOAEs).”

 “The suppression of OAE obtained by presenting noise to the non-test ear is known as the contralateral suppression of OAE.”

 The authors refer to review by Guinan, but he uses term of inhibition not suppression and it is directly stated why in the referenced paper. This should be at least commented.

**Authors reply: **Citations added to the manuscript 

 Method:

**Reviewer comment:** How much caffeine was in the coffee? Please provide information of the amount of caffeine used for the experiments.

**Authors reply: **The caffeine content ranges from 2.2 to 3.1%. Thus, the amount of caffeine ranged from 27 to 40 mg per cup of coffee. Same information is added to the manuscript.  

**Reviewer comment: **It would be good if the authors evaluated effect of coffee by some other test, to show that coffee influenced the subject at all, e.g. in studies of effect of attention on OAEs the attention level is evaluated by counting of stimuli or by evoked potentials, and then OAE analyses are made.

**Authors reply:** We agree with your recommendation, but other tests were not performed during the study. We acknowledge the same in discussion 

**Reviewer comment**: How the authors evaluated the middle ear muscle reflex (MEMR)? Several studies point to MEMR interaction with experiments based on contralateral suppression of OAEs. The authors should check the MEMR influence or at least mention lack of it as the limitation of the study.

**Authors reply:** The middle ear muscle reflex was evaluated by measuring the acoustic reflex threshold for white noise. GSI Tympstar Pro immittance meter was used for measuring the reflex threshold for white noise. The lowest intensity at which a change in admittance was noted (referred as ‘reflex threshold’) was considered as an evidence of middle ear muscle reflex. In the present study the white noise was presented below the reflex threshold, during the measurement of contralateral suppression of TEOAE. Similar approach has been employed by several studies measuring the contralateral suppression of TEOAE.  

**Reviewer comment:** Why are OAE parameters calculated by some program, and not taken from the ILO system?

**Authors reply:** During the study period, the computer used for recording and storing the OAE data failed. Data was not lost, as data files were copied to another computer. We could not find a way to load the data files back to ILO software platform for the purpose of data analysis. Thus, OAE parameters was calculated using EchoMaster software.  

**Reviewer comment: **Provide some rationale for using 3 dB SNR criterion while studies of reliability of OAE suppression recommend using at least 6 dB or even more.

**Authors reply: **We have reanalyzed the data and presented results using 6 dB SNR criterion.  

 Results:

**Reviewer comment: **Please provide error bars on figures 4 and 5.

**Authors reply: **Error bars (SD) added to figure 4. Data showed in figure 5 is same as figure 4.

**Reviewer comment: **Maybe the authors could select smaller dataset with OAEs of SNR > 6dB?

**Authors reply: **We have reanalyzed the data and presented results using 6 dB SNR criterion.  

 Discussion:

**Reviewer comment: **Study is based on TEOAEs so the comparisons to DPOAEs should be limited.

**Authors reply:** As studies related to TEOAEs are not available in literature, DPOAEs are cited for the purpose of comparison.  

 Please compare results with other studies of suppression on same equipment, especially recent studies of reliability/variability. Even if there are no studies of coffee on suppression of OAEs, there are some studies of reliability of TEOAEs measured by the same equipment. These are very good basis for comparison, and for reference what change in TEOAE suppression would show the significant effect.

 References:

**Reviewer comment:** Mistakes – references to papers in which there is no referenced information. For example: “Further, the OAEs elicited in response to short-duration stimuli such as clicks and tone-bursts are known as transient evoked OAEs (TEOAEs).” which cites Kemp 1978. At this time Kemp did not used the name of TEOAEs.

**Authors reply:** Citation changed and error corrected
